# 

**Published:** 1875-04

**Authors:** 


CONTENTS.

| | PAGE | | PAGE |
|---|---|---|---|
| Woman's Apparel | 101 | Clean Teeth | 121 |
| A Reform Needed | 103 | The Guillotine | 122 |
| Pauperism and Crime | 104 | Cheese as Food | 123 |
| Our Summer at Maplewood. | | Sweetening the Food of Animals | 124 |
| Chap. III. Still dabbling in dough | 105 | Heliotype | 125 |
| Tea | 113 | Home Adornment | 126 |
| Visiting the Sick | 114 | Our Dumb Animals | 127 |
| Arsenic in Wall-Paper | 115 | An Important Question | 128 |
| Personal and Organized Charity | 116 | Milk Granules | 129 |
| The House of Steinway | 117 | Brain Disease | 130 |
| Privies and Vaults | 118 | Water in Pain | 131 |
| Electricity and Life | 119 | The "Utility." | 132 |
| Genuine Charity | 120 | Deformed Feet | 133 |

